# Salivary epidermal growth factor correlates with hospitalization length in rotavirus infection

**DOI:** 10.1186/s12879-017-2463-0

**Published:** 2017-05-30

**Authors:** J. Gómez-Rial, M. J. Curras-Tuala, C. Talavero-González, C. Rodríguez-Tenreiro, L. Vilanova-Trillo, A. Gómez-Carballa, I. Rivero-Calle, A. Justicia-Grande, J. Pardo-Seco, L. Redondo-Collazo, A. Salas, F. Martinón-Torres

**Affiliations:** 1 Grupo de Investigación en Genética, Vacunas, Infecciones y Pediatría (GENVIP), Hospital Clínico Universitario de Santiago de Compostela, Santiago de Compostela, Galicia Spain; 2 Laboratorio de Inmunología, Servicio de Análisis Clínicos, Hospital Clínico Universitario de Santiago de Compostela, Santiago de Compostela, Galicia Spain; 3 Translational Pediatrics and Infectious Diseases, Department of Pediatrics, Hospital Clínico Universitario de Santiago de Compostela, Santiago de Compostela, Galicia Spain; 4 Unidade de Xenética, Departamento de Anatomía Patolóxica e Ciencias Forenses, Instituto de Ciencias Forenses, Facultade de Medicina, Universidade de Santiago de Compostela, Santiago de Compostela, Galicia Spain; 5 GenPob Research Group, Instituto de Investigaciones Sanitarias (IDIS), Hospital Clínico Universitario de Santiago, Santiago de Compostela, Galicia Spain

**Keywords:** Biomarkers, EGF, IFI27, Rotavirus infection

## Abstract

**Background:**

The *IFI27* interferon gene expression has been found to be largely increased in rotavirus (RV)-infected patients. *IFI27* gene encodes for a protein of unknown function, very recently linked to epidermal proliferation and related to the epidermal growth factor (EGF) protein. The EGF is a low-molecular-weight polypeptide that is mainly produced by submandibular and parotid glands, and it plays an important physiological role in the maintenance of oro-esophageal and gastric tissue integrity.

Our aim was to determine salivary EGF levels in RV-infected patients in order to establish its potential relationship with *IFI27* increased expression and EGF-mediated mucosal protection in RV infection.

**Methods:**

We conducted a prospective comparative study using saliva samples from 27 infants infected with RV (sampled at recruitment during hospital admission and at convalescence, i.e. at least 3 months after recovery) and from 36 healthy control children.

**Results:**

Median (SD) EGF salivary concentration was 777 (529) pg/ml in RV-infected group at acute phase and 356 (242) pg/m at convalescence, while it was 337 (119) pg/ml in the healthy control group. A significant association was found between EGF levels and hospitalization length of stay (*P*-value = 0.022; r^2^ = −0.63).

**Conclusions:**

The salivary levels of EGF are significantly increased during the acute phase of natural RV infection, and relate to length of hospitalization. Further assessment of this non-invasive biomarker in RV disease is warranted.

## Background

Rotavirus (RV) infection is the most common cause of severe diarrhoea in children and may cause serious dehydration that usually requires hospitalization [1]. Predominantly, but not exclusively, RV infects the terminally differentiated enterocytes in the small intestine and it induces mucosal damage, villus atrophy and necrosis of epithelial cells [2]. RV infection alters the function of the small intestinal epithelium, resulting in characteristic diarrhoea, secondary to enterocyte destruction.

**Table 1:** Demographic characteristics of the study patients expressed as mean (SD)

Variable	RV acute-phase	RV convalescence	Healthy controls
Demographic data			
*n*	27	23	36
Age [months (SD)]	12.5 (1–40)	18 (5–47)	7 (6–7)
Sex (male:female)	16:11	14:9	18:18

**Table 2:** Clinical characteristics of patients in acute phase expressed as mean (SD)

Clinical data	RV acute-phase
Vesikari’s score	11.07 (3.485)
Length of stay in hospital (days)	5.750 (3.026)
Temperature (°C)	38.70 (0.622)
Number of vomiting episodes per day	3.179 (4.738)
Duration of vomiting (days)	2.037 (1.931)
Number of stools per day	4.929 (5.018)
Duration of diarrhoea (days)	4.571 (2.168)
Level of dehydration	1.607 (1.100)

**Fig. 1:**
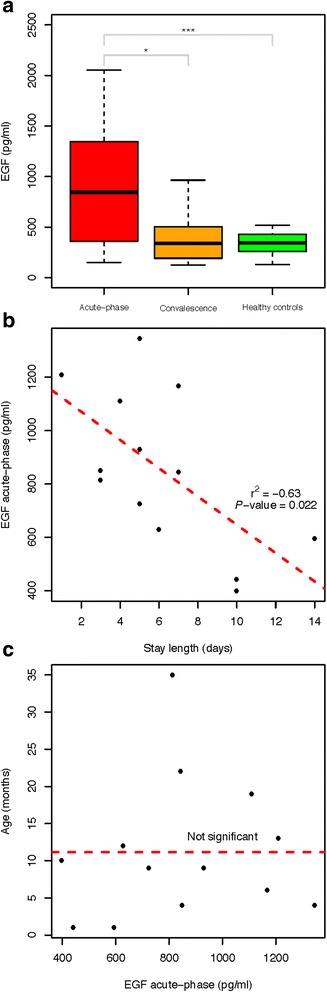
**a** Values of EGF measured in saliva from RV-infected patients during acute phase (*n* = 27) and convalescence (*n* = 23), and in healthy controls (*n* = 36). Saliva samples were tested for EGF using multiplex cytokine analysis as described in the text. Results are presented as median and interquartile range EGF levels in pg/ml. A non-parametric Wilcoxon test was used to determine statistical significance between acute-phase and convalescence samples. Mann-Whitney tests were used for comparison between acute-phase and healthy controls. *P*-values indicated on graphs are <0.05 (*) and <0.0001 (***). **b** Association between salivary EGF level and length of hospitalization stay. Salivary EGF levels and symptom scores for 27 RV-infected patients were analysed as described in the text. No other significant association was detected. Spearman’s rank correlation coefficient (r^2^) is presented (*P-value* = 0.022). **c** Age and salivary EGF levels. No association was observed between EGF level and age

There are currently not reliable specific biomarkers for RV infection. Identifying new correlates of disease activity may help in designing new treatments and more efficacious RV vaccines. Use of saliva as a proxy indicator of intestinal immunity has been proposed as a non-invasive source of biomarkers [3]. Salivary IgA has been recently suggested as a correlate for protection for norovirus intestinal infection [4].

In a recent whole blood transcriptomic analysis of RV-infected patients, we reported a 23.7 fold increase in the expression of the *IFI27* gene, an interferon-related gene with unknown function in the RV infection [5]. Moreover, this gene has recently been associated to the epidermal growth factor (EGF) and involved in proliferation of epithelia [6]. EGF, also known as epithelial growth factor, is a single chain polypeptide secreted by the submandibular salivary glands and other exocrine intestinal glands. In vivo actions of EGF in the gastrointestinal tract include promoting wound healing and tissue repair, inducing epithelium proliferation and promoting differentiation [7]. A significant contribution of salivary EGF to the maintenance of the integrity of the oesophageal mucosa has been demonstrated in experimental and clinical settings [8]. Patients with low salivary EGF levels are predisposed to severe oesophageal damage by gastro-oesophageal reflux [9].

The aim of the present study was to measure the levels of EGF in saliva from acute-phase RV-infected children and to assess its potential relationship with disease course and severity.

## Methods

### Patients and controls

This is a prospective single-centre case-control study consisting of the collection of saliva samples and clinical data from pediatric patients admitted to hospital due to a RV infection, and age- and gender-matched healthy controls. A total of 27 patients with RV infection were prospectively recruited at the Pediatric Department of Hospital Clínico Universitario of Santiago de Compostela (Spain) during the period 2013–2014, all of them hospitalized with acute gastroenteritis and with a positive RV antigen detected in stool. Demographic and clinical data, including fever, vomiting, diarrhoea, dehydration and total severity scores during entire period of illness were obtained. During the same period, 36 healthy controls were enrolled in the study.

Saliva samples were collected at recruitment on admission to the hospital (acute phase) and at convalescence (at least 90 days after infection) for the RV-infected group. A single sample was obtained at recruitment for healthy controls group.

The saliva sample was obtained from unstimulated sublingual saliva using oral swabs (Whatman; Maidstone, Kent, UK) placed under the tongue for 5 min. The swabs were eluted in 0.4 ml of phosphate buffered saline (PBS), and then centrifuged at 800 g for 10 min to remove mucin and epithelial cells. Supernatants were stored at −30 °C prior to analysis.

#### Clinical data collection

Temperature, number of vomiting episodes per day and duration of vomiting, the severity of diarrhoea (numbers of stools per day, duration of diarrhoea, and level of dehydration), length of hospital stay, and total symptoms scores measured using Vesikari’s score were registered in RV-infected subjects.

#### Detection of EGF in saliva samples

EGF detection assay was carried out using Milliplex Map human cytokine detection kit (Merck Millipore, Billerica, MA), following manufacturer indications. Assays were carried out on a Luminex™ 200 platform.

#### Statistical analysis

Data are expressed as median and interquartile range. Statistical analyses were performed using the statistical software R v.3.1.1 (http://www.r-project.org). Outliers were identified as those values falling above or below boxplot whiskers, and eliminated from further analysis. Non-parametric statistics were used for analysis because data were not normally distributed in the cohort of cases. Wilcoxon rank-sum tests were used for comparison between acute-phase versus convalescence samples and versus healthy controls. Spearman’s rank correlation coefficients were used to quantify the association between EGF concentration and disease clinical parameters. Reported *P*-values were below the Bonferroni significant threshold.

## Results

Patients in the RV-infected group ranged in age from 1 to 40 months (median of 12.5 months) at recruitment. Healthy control children ranged in age from 6 to 7 months (median of 7 months). Demographic and clinical characteristics are summarized in Tables 1 and 2.

Median levels for EGF in saliva from acute-phase RV-infected patients were 777 (SD = 529) pg/ml. Salivary EGF levels where significantly lower in convalescent patients (356 pg/ml; SD = 242; Wilconxon test, *P*-value = 0.015) and healthy controls (337 pg/ml; SD = 119; Wilconxon test, *P*-value = 0.001474); Fig. 1a. These variations in EGF levels were not age-related (Fig. 1b).

When assessing possible correlations between EGF levels and clinical variables in RV infected subjects, a significant negative correlation was found between salivary EGF concentration and length of hospital stay (*P*-value = 0.022; *r* ^*2*^ = −0.63). We did not find any other significant association between EGF levels and other clinical parameters of disease (Fig. 1c).

## Discussion

In a previous whole blood transcriptomic analysis [5] carried out in RV-infected patients vs control children, a remarkable over-expression of *IFI27* gene was reported. *IFI27* belongs to the family of interferon inducible genes, and recently it has been associated to epidermal proliferation and EGF [6]. In addition, *IFI27* was previously identified as a marker of epithelial cancers and psoriatic lesions [10] and related to systemic lupus erythematosus (SLE) [11]. However, the exact role of *IFI27* in the RV infection physiopathology is still unknown, although based on our results we can suggest that RV infection might activate an interferon-mediated mechanism of mucosal recovery after infection damage through a pathway mediated by *IFI27*. The observed association between *IFI27* expression and EGF requires a more in-depth analysis, and the mechanistic approach needs to be clarified.

EGF is linked to epithelia regeneration after mucosal lesions, promoting mucosal wound healing and tissue repair [12]. Previous studies in neonatal pigs infected with RV already pointed to a beneficial role of high physiological levels of EGF in stimulating recovery of epithelium in the small intestine following infection [13]. EGF application in the mucosa can promote intestinal proliferation and improve restoration after intestinal injury, and it might be an effective treatment against intestinal ischemia-reperfusion injury in rats [14]. The hypothesis of EGF supplementation after intestinal injury as a potential treatment for improving outcome in patients has been put forward by other authors based on animal studies [15]. This finding might lay the ground for a new recovery treatment after RV infection.

Our present study shows that salivary levels of EGF are increased during natural RV infection and that they may correlate with the severity of the disease in terms of length of hospital stay. The over-expression of EGF levels in infected patients might suggest a host recovery response to the damage induced by RV in the intestinal mucosa. The disruption in the normal homeostasis of mucosa would induce the production of EGF, also expressed in the submandibular salivary glands, in order to restore the integrity of mucosa after infection.

Interestingly, we found that the higher the levels of EGF in acute-phase samples from RV-infected patients upon admission, the shorter the length of stay in hospital. It could be argued that the greater level of EGF reflects a more powerful response of mucosal homeostasis, thus facilitating a faster recovery, and a reduction in the length of hospitalization. This association, if prospectively confirmed, might allow to make predictions on recovery of mucosal integrity and hospitalization length with a simple and non-invasive methodology. The use of saliva as a non-invasive proxy for intestinal immunity after enteric infection opens the door to the search for new specific biomarkers for RV infection that might correlate with clinical parameters of disease.

One limitation of the present study is the limited number of individual analysed, however the novelty of our findings merits consideration and further confirmatory studies. Another limitation derives from the different localization of previous gene expression analyses (performed in whole blood), and our EGF quantification (performed in the epithelial mucosa).

## Conclusions

In conclusion, RV infection induces an increased production of EGF by submandibular glands that could be associated to *IFI27* gene over-expression. Moreover, increased salivary EGF concentrations were found to be associated with length of hospitalization. Further studies to corroborate our findings are needed. In the meantime, we can postulate saliva as a good proxy to advance in our knowledge of RV infection.

## Abbreviations

EGF: Epidermal growth factor
IFI27: Interferon-alpha inducible protein 27
RV: Rotavirus
SLE: Systemic Lupus Erythematous
